# Investigation of the clinical significance and pathological features of lanthanum deposition in the gastric mucosa

**DOI:** 10.1186/s12876-020-01545-z

**Published:** 2020-11-23

**Authors:** Shinya Nishida, Kazuhiro Ota, Kimiaki Hattori, Taro Iwatsubo, Shimpei Kawaguchi, Yuichi Kojima, Toshihisa Takeuchi, Tamaki Maeda, Masahiro Sakaguchi, Kazuhide Higuchi

**Affiliations:** 1 Second Department of Internal Medicine, Osaka Medical College, 2-7, Daigaku-machi, Takatsuki, Osaka 569-8686 Japan; 2 Department of Internal Medicine, Moriguchi Keijinkai Hospital, Moriguchi, Osaka Japan; 3 Department of Pathology, Moriguchi Keijinkai Hospital, Moriguchi, Osaka Japan; 4 Department of Pathology, Osaka Medical College, Takatsuki, Osaka Japan

**Keywords:** Lanthanum, Phosphate binder, Histiocytic infiltration, Whitish lesion

## Abstract

**Background:**

There are often specific endoscopic findings caused by deposition of lanthanum (La) in the gastric mucosa of patients taking lanthanum carbonate (LaC), a novel phosphate binder for patients on hemodialysis. We conducted a retrospective study to investigate the clinical significance of La deposition in the gastric mucosa, and the association between endoscopic features and histologic findings in the same population.

**Methods:**

We compared background factors in patients taking LaC with and without La deposition in their gastroscopic biopsy specimen. We also investigated the relationship between gastric endoscopic biopsy specimens with La deposition and the concurrent endoscopic images.

**Results:**

There was a significant difference in the total dose of LaC between the La-positive and La-negative groups (990 g [180–3150 g] vs. 480 g [225–1328 g]; *p* = 0.013). In 27 biopsy specimens with specific whitish mucosa, 10 showed mild histiocytic infiltration and 17 showed severe infiltration. In contrast, among 24 specimens with non-whitish mucosa, 5 showed no histiocytic infiltration, 10 showed mild infiltration, and 9 showed severe infiltration. There was a significant relationship between endoscopic features and the degree of histiocytic infiltration (*p* = 0.026).

**Conclusions:**

We demonstrated that La deposition in the gastric mucosa depended on the total dose of LaC and was not affected by background factors. The specific endoscopic features of La deposition are associated with the infiltration of histiocytes, which represents the body’s normal response to foreign bodies.

*Trial registry* The protocol was registered in the University Hospital Medical Information Network Clinical Trial Registry (UMIN000038929, https://upload.umin.ac.jp/cgi-open-bin/ctr/ctr_view.cgi?recptno=R000044393).

## Background

The number of dialysis patients in Japan reached 329,609 in 2016 [1]. For dialysis patients, hyperphosphatemia is one of the critical complications because it significantly increases cardiovascular disease and mortality. To control the blood concentration of phosphoric acid, several types of oral phosphate binders have been developed, including lanthanum carbonate (LaC) (Fosrenol®; Shire Pharmaceuticals, Hampshire, UK), which has been authorized by the Ministry of Health, Labor and Welfare in Japan. LaC is the first non-calcium, non-resin phosphate binder for hyperphosphatasemia in patients with chronic kidney disease on hemodialysis. The use of LaC has increased in Japan since its launch in 2009 as it has fewer side effects than conventional medicines. Recently, it was reported that there are specific endoscopic findings caused by deposition of lanthanum (La) in the gastric mucosa of patients taking LaC [2].

We previously reported that the daily dose and total dose of LaC were significantly correlated with La deposition in the gastroduodenal mucosa [3]. However, it is still unknown why these phenomena appear in LaC users and what subsequent difficulties, if any, appear in patients with La deposition in the gastrointestinal mucosa. Although it was shown that patients on hemodialysis with anemia are at a high risk of small intestinal lesions in our previous study [4], the relationships between La deposition and other factors were not investigated.

Murakami et al. investigated seven cases with the specific endoscopic features of La deposition in the gastric mucosa and described the whitish lesions as follows: an annular whitish mucosa, diffuse whitish mucosa, and whitish spots in the gastric mucosa [5]. Although the endoscopic biopsy specimens from the above endoscopic features revealed La deposition histologically, it was uncertain whether these whitish lesions were pathologically consistent with La deposition.

We therefore conducted the two following clinical retrospective investigations: (1) comparative analysis of the clinical features of LaC users with and without La deposition in endoscopic biopsy specimens from gastric mucosa and (2) histological identification of the cause of the whitish lesions using biopsy specimens with La deposition by comparing the histological features of the endoscopic findings.

## Methods

### Patient population and study design

#### Study 1

This retrospective observational investigation included patients on hemodialysis taking LaC from whom biopsies from the gastric mucosa were taken via gastric endoscopy at Moriguchi Keijinkai Hospital between April 2014 and August 2019. Most dialysis patients in the Moriguchi Keijinkai Hospital are advised to undergo esophagogastroduodenoscopy regularly to screen for malignant tumors by their attending physician even if they are asymptomatic. An endoscopist judges each case based on its individual characteristics before performing endoscopic biopsy. The aims of biopsy varied: searching for malignant tumors, confirming benign lesions, and determining the cause of unknown lesions (including whitish mucosal lesion by La deposition). All biopsy specimens, which were fixed with 10% formalin and embedded in paraffin, were stained with hematoxylin and eosin. In the histological evaluation of biopsy specimens, the crystalloids dyed with eosin were diagnosed as La deposition based on a previous report [3]. Our main objective was to reveal the clinical significance of La deposition in the gastric mucosa by comparing the clinical features of patients with and without La deposition in endoscopic biopsy specimens from the gastric mucosa. The characteristics compared were as follows: sex, age, the degree of gastric mucosal atrophy, the primary disease at time of dialysis introduction, the dialysis period, the period of taking LaC, the daily dose of LaC, the total dose of LaC, blood test findings (calcium, inorganic phosphorus, total protein, albumin, total cholesterol, high density lipoprotein-cholesterol, triglyceride, white blood cell count, and hemoglobin), and concomitant drugs (non-steroidal anti-inflammatory drugs [NSAIDs] including low-dose aspirin, proton pump inhibitors [PPIs], histamine H2 receptor antagonists [H2RAs], and gastric mucoprotective drugs). All items were collected two weeks before and after endoscopic biopsy.

In addition, we compared the group of patients taking LaC with the control group of dialysis patients; the abovementioned patients taking LaC with and without gastric La deposits, and the control group of dialysis patients who were not taking LaC and underwent endoscopic biopsy in the same period.

#### Study 2

**Fig. 1:**
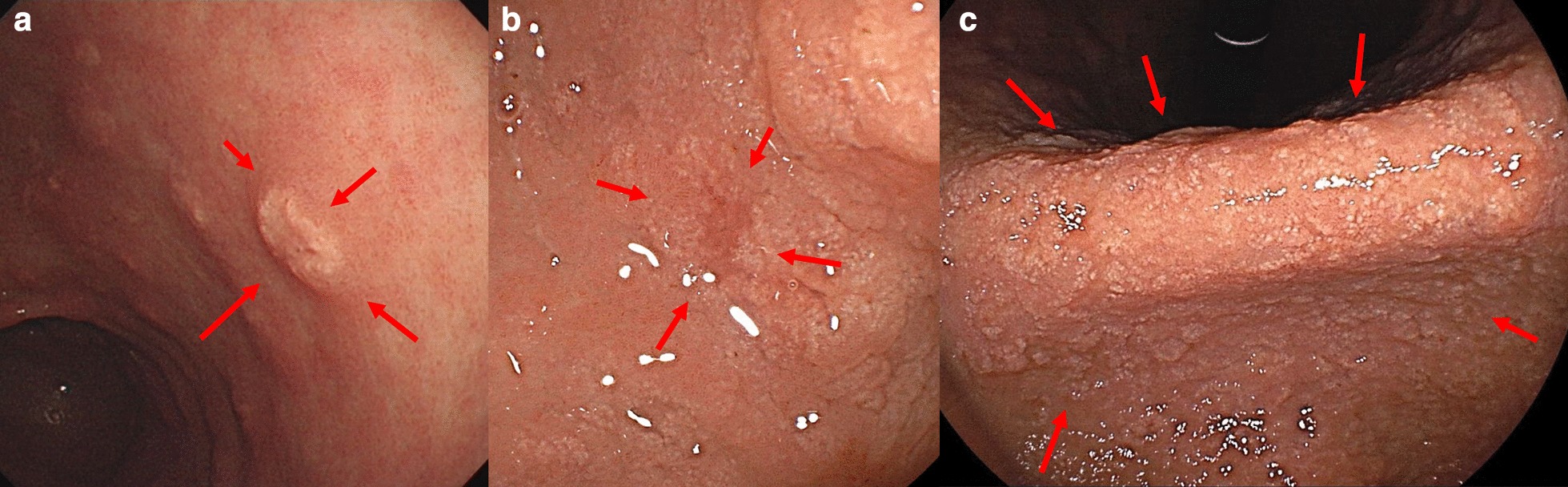
Specific endoscopic feature (red arrows) of La deposition in the gastric mucosa at the endoscopic biopsy sites. **a** Whitish spot, **b** annular whitish mucosa and **c** diffuse whitish mucosa

This retrospective investigation was performed using the gastric endoscopic biopsy specimens with La deposition as well as the concurrent endoscopic images. First, we confirmed the presence or absence of whitish lesions—the specific endoscopic feature of La deposition—in the gastric mucosa at the endoscopic biopsy sites (Fig. 1). In this study, we determined the specific whitish mucosa of the biopsy site in the following cases: when an endoscopist clearly recognized the whitish lesions and biopsied them, or when a biopsy site unexpectedly contained a white lesion. Two independent gastrointestinal endoscopists (S.N. and K.O.) performed these evaluations retrospectively. When opinions differed between the endoscopists, a third independent gastrointestinal endoscopist (Y.K.) was consulted. Second, for each biopsy specimen, we evaluated the amount of deposited La and the degree of histiocytic infiltration, which are the characteristic findings for diagnosis of La deposition. These histological evaluations were performed by two pathologists (K.H. and T.M.). Finally, we contrasted the endoscopic features and histological findings and investigated the pathological significance of the whitish lesions as specific findings of La deposition.

### Statistical analysis

Significant differences between groups were determined using the Mann–Whitney *U* test for continuous or categorical variables and the Pearson’s chi-square test for binary variables. The trends in binomial proportions across the levels of a single variable were determined using the Cochran-Armitage trend test. All reported p-values were two-sided, and values < 0.05 were considered statistically significant. All calculations were performed with JMP Pro 14 software (SAS Institute Inc., Cary, NC, USA).

## Results

### Study 1

This analysis was performed on 42 patients who were on hemodialysis and taking LaC medication and who underwent gastric endoscopy and endoscopic biopsy from the gastric mucosa at Moriguchi Keijinkai Hospital during the study period. The number of patients with La deposition in the gastric mucosa (La-positive group) was 23, and the number without La deposition was 19 (La-negative group). There was a significant difference between the two groups regarding the total dose of LaC (La-positive group, 990 g [180–3150 g]; La-negative group, 480 g [225–1328 g]; *p* = 0.013, Mann–Whitney *U* test). Other variables, including the daily dose of LaC, were not significantly different in the two groups; however, there was some missing blood test data (Table 1).

**Table 1:** Patient characteristics in Study 1

Patient characteristics	La deposition (+), n = 23	La deposition (−), n = 19	Odds ratio (95% CI)	*p* value
Male sex, n (%)	14 (60.9%)	13 (68.4%)	1.39 (0.39–5.01)	0.258
Age, median (range)	72 (47–82)	68 (45–83)	–	0.909
Gastric mucosal atrophy (mild, medium, severe)*, n (%)	5 (21.7%), 7 (30.4%), 11 (47.8%)	7 (36.8%), 3 (15.8%), 9 (47.4%)	–	0.413
Primary diseases of dialysis (diabetes, hypertension, nephritis), n (%)	8 (34.8%), 3 (13.4%), 12 (52.2%)	10 (52.6%), 2 (10.5%), 7 (36.8%)	–	0.504
Years since start of dialysis, median (range)	7.32 (0.70–26.4)	5.83 (1.57–14.2)	–	0.092
Months of oral use of LaC, median (range)	34 (4–70)	28 (10–72)	–	0.206
Daily dose of LaC, mg, median (range)	1000 (500–1500)	750 (250–1500)	–	0.104
Total dose of LaC, g, median (range)	990 (180–3150)	480 (225–1328)	–	*0.013*
Ca, mg/dL, median (range) (number of missing data: 4)	8.9 (8–10.2)	8.4 (7.2–9.9)	–	0.084
IP, mg/dL, median (range) (number of missing data: 7)	5.5 (3.5–8.1)	5.3 (3.1–8.3)	–	0.586
TP, g/dL, median (range) (number of missing data: 8)	6.4 (4.8–7.9)	6.35 (4.3–7.3)	–	0.479
Albumin, g/dL, median (range) (number of missing data: 7)	3.5 (2.7–4.0)	3.6 (2.4–4.0)	–	0.752
Total cholesterol, mg/dL, median (range) (number of missing data: 10)	166 (103–203)	151 (88–240)	–	0.454
HDL–cholesterol, mg/dL, median (range) (number of missing data: 13)	47 (24–67)	47 (18–97)	–	0.790
Triglyceride, mg/dL, median (range) (number of missing data: 9)	103 (50–336)	94.5 (35–198)	–	0.649
WBC, /µL, median (range) (number of missing data: 6)	5460 (3370–8300)	6535 (2650–12,470)	–	0.133
Hemoglobin, g/dL, median (range) (number of missing data: 5)	10.5 (7.3–12.5)	10.55 (7.3–12.8)	–	0.976
NSAIDs (including low–dose aspirin), n (%)	10 (43.5%)	10 (52.6%)	0.69 (0.20–2.35)	0.554
PPIs, n (%)	17 (73.9%)	17 (89.5%)	0.33 (0.06–1.89)	0.201
PPIs + H2RAs, n (%)	21 (91.3%)	17 (89.5%)	1.24 (0.16–9.71)	0.841
Gastric mucoprotective drugs, n (%)	9 (39.1%)	4 (21.1%)	2.41 (0.60–9.63)	0.207

La, lanthanum; CI, confidence interval; LaC, lanthanum carbonate; Ca, calcium; IP, inorganic phosphorus; TP, total protein; WBC, white blood cell; NSAID, non-steroidal anti-inflammatory drug; PPI, proton pump inhibitor; H2RA, histamine H2 receptor antagonist

*The degree of gastric mucosal atrophy is classified into mild, medium, or severe according to the Takemoto-Kimura classification [13]. Mild: C-1 and C-2, medium: C-3 and O-1, severe: O-2 and O-3

Italic font represents statistical significance

There was a significant difference in years since start of dialysis between the LaC-taking group (n = 42) and the control dialysis group (n = 42). There were no significant differences in other characteristics between the two groups (Table 2).

**Table 2:** Comparison of the LaC-taking patients and the control dialysis patients

Patient characteristics	LaC–taking patients, n = 42	Control dialysis patients, n = 42	Odds ratio (95% CI)	*p* value
Male sex, n (%)	15 (35.7%)	20 (47.6%)	0.61 (0.25–1.47)	0.269
Age, median (range)	68 (45–83)	70 (35–86)	–	0.400
Gastric mucosal atrophy (mild, medium, severe)*, n (%)	12 (28.6%), 10 (23.8%) 20 (47.6%)	14 (33.3%), 10 (23.8%), 18 (42.9%)	–	0.879
Primary diseases of dialysis (diabetes, hypertension, nephritis), n (%)	18 (42.9%), 5 (11.9%), 19 (45.2%)	13 (31.0%), 7 (16.7%), 22 (52.4%)	–	0.507
Years since start of dialysis, median (range)	6.12 (0.7–26.4)	3.58 (0.002–12.5)	–	*0.0002*
Ca, mg/dL, median (range)	8.75 (7.2–10.2)	8.9 (6.1–10.3)	–	0.300
IP, mg/dL, median (range)	5.5 (3.1–8.3)	4.9 (1.9–7.5)	–	0.320
TP, g/dL, median (range)	6.4 (4.3–7.9)	6.6 (3.9–7.9)	–	0.184
Albumin, g/dL, median (range)	3.5 (2.4–4.0)	3.5 (2.5–4.7)	–	0.362
Total cholesterol, mg/dL, median (range)	158.5 (88–240)	160 (93–303)	–	0.402
HDL–cholesterol, mg/dL, median (range)	47 (18–97)	49.5 (23–78)	–	0.601
Triglyceride, mg/dL, median (range)	97 (35–336)	92.5 (36–271)	–	0.721
WBC, /µL, median (range)	5720 (2650–12,470)	5210 (2800–11,800)	–	0.068
Hemoglobin, g/dL, median (range)	10.5 (7.3–12.8)	10.6 (7.1–12.4)	–	0.709
NSAIDs (including low–dose aspirin), n (%)	18 (42.9%)	10 (23.8%)	2.4 (0.94–6.12)	0.064
PPIs, n (%)	34 (81.0%)	32 (76.2%)	1.32 (0.47–3.79)	0.595
PPIs + H2RA, n (%)	38 (90.5%)	35 (83.3%)	1.9 (0.51–7.05)	0.332
Gastric mucoprotective drugs, n (%)	13 (31.0%)	6 (14.3%)	2.79 (0.91–7.95)	0.068

La, lanthanum; CI, confidence interval; LaC, lanthanum carbonate; Ca, calcium; IP, inorganic phosphorus; TP, total protein; WBC, white blood cell; NSAID, non-steroidal anti-inflammatory drug; PPI, proton pump inhibitor; H2RA, histamine H2 receptor antagonist

*The degree of gastric mucosal atrophy is classified into mild, medium, or severe according to the Takemoto-Kimura classification [13]. Mild: C-1 and C-2, medium: C-3 and O-1, severe: O-2 and O-3

Italic font represents statistical significance

### Study 2

The investigation was performed on 51 biopsy specimens from 23 patients with La deposition in the gastric mucosa and the endoscopic images corresponding to each biopsy site. The 27 biopsy specimens with the characteristic whitish gastric mucosa showed La deposition. La deposition was also detected on the 24 biopsy specimens with non-whitish mucosa.

**Fig. 2:**
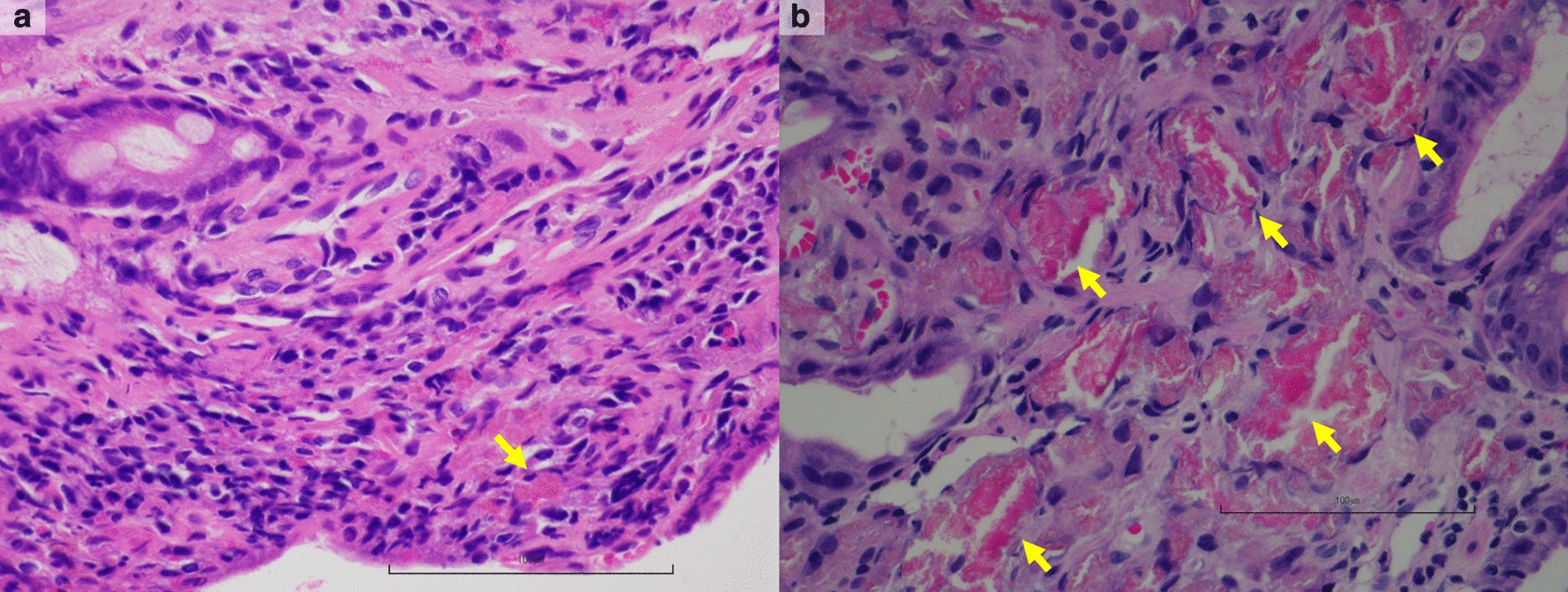
Histologic features of La crystalloid (hematoxylin–eosin stain). The bar shows 100 µm. **a** Whole specimen from the gastric mucosa with very small fragments of La crystalloid materials (yellow arrows). There are one or two small fragments of La crystalloid in the slice of the specimen. **b** Whole specimen from the gastric mucosa with more La crystalloid materials (yellow arrows)

**Fig. 3:**
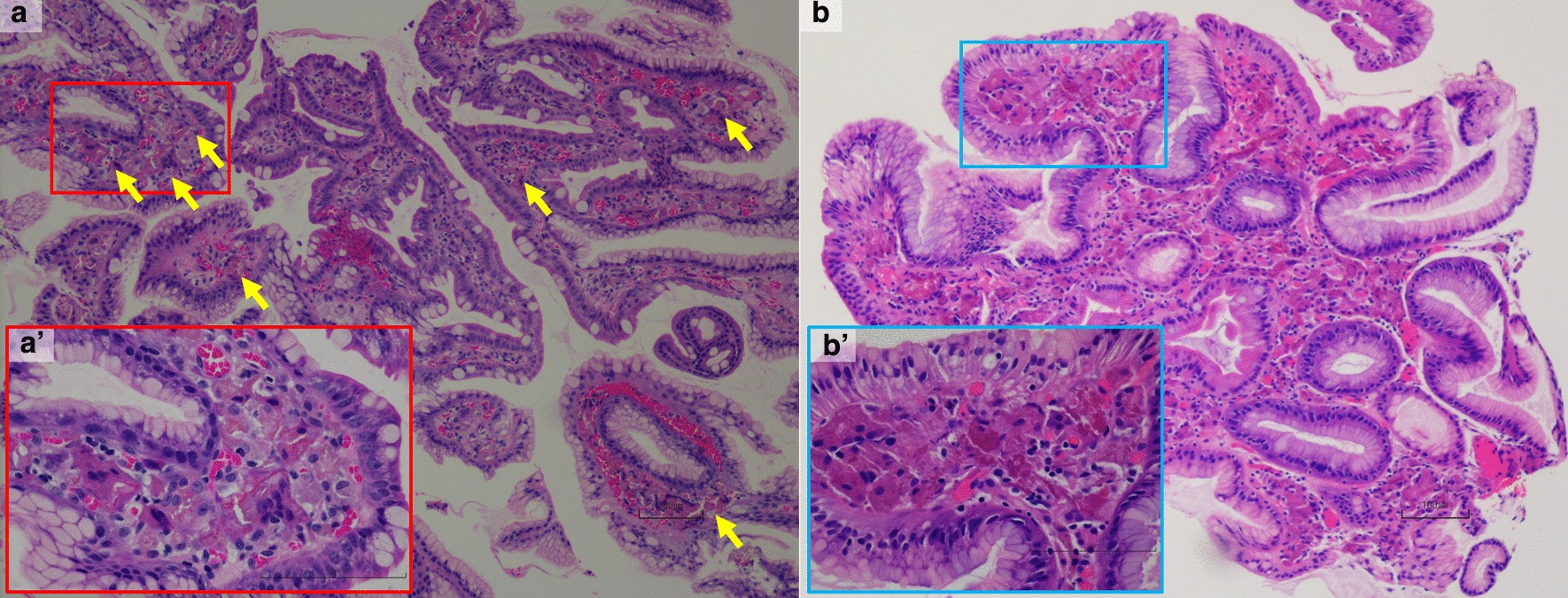
Histologic features of histiocytes aggregating (hematoxylin–eosin stain). The bar shows 100 µm. **a** Less than 30% of the lamina propria is occupied by histiocytes aggregating in the gastric mucosa (yellow arrows). **a′** The enlarged view of the red square in **a**. **b** More than 80% of the lamina propria is occupied by histiocytes aggregating in the gastric mucosa. **b′** The enlarged view of the blue square in **b**

We classified the patients into two groups according to the amount of La deposition in the biopsy specimen. One or two small fragments of La crystalloid were observed in the slice of 31 specimens (Fig. 2a). The other 20 specimens showed three or more crystalloids (Fig. 2b). In 20 specimens, less than 30% of the lamina propria was occupied by histiocytes (mild infiltration, Fig. 3a), and more than 80% of the lamina propria was occupied by histiocytes in 26 specimens (severe infiltration, Fig. 3b). The other five specimens showed no histiocyte occupation. This assessment of histiocytic infiltration was classified by the pathologists’ subjective opinions.

Of 27 biopsy specimens with the specific whitish mucosa, 14 showed a small amount of La deposition, while 13 showed a large amount. In contrast, in the 24 specimens with non-whitish mucosa, 17 showed a small amount of La deposition and 7 showed a large amount. There was no significant relationship between the endoscopic features and the amount of La deposition (*p* = 0.166, Pearson’s chi-square test; Table 3). In the 27 biopsy specimens with specific whitish mucosa, 10 showed mild histiocytic infiltration and 17 showed severe infiltration. In contrast, among the 24 specimens with non-whitish mucosa, 5 showed no histiocytic infiltration, 10 showed mild infiltration, and 9 showed severe infiltration. There was a significant relationship between the endoscopic features and the degree of histiocytic infiltration (*p* = 0.026, Pearson’s chi-square test; *p* = 0.013, Cochran-Armitage trend test; Table 4).

**Table 3:** The relationship between the endoscopic features and the amount of La deposition

Amount of La crystalloid materials	One or two small fragments of La crystalloid	Three or more La crystalloids
Specific whitish mucosa+ 27 biopsy specimens	14 (51.9%)	13 (48.2%)
Specific whitish mucosa− 24 biopsy specimens	17 (70.8%)	7 (29.2)

Data are presented as number (percentage)

Pearson’s chi-square test: *p* = 0.166

**Table 4:** The relationship between endoscopic features and degree of histiocytic infiltration

Histiocytic infiltration	None	Mild	Severe
Specific whitish mucosa+ 27 biopsy specimens	0	10 (37.0%)	17 (63.0%)
Specific whitish mucosa− 24 biopsy specimens	5 (20.8%)	10 (41.7%)	9 (37.5%)

Data are presented as number (percentage)

Pearson’s chi-square test: *p* = 0.026, Cochran-Armitage trend test: *p* = 0.013

## Discussion

To the best of our knowledge, this retrospective study is the first to investigate the clinical significance of La deposition in the gastric mucosa (as in Study 1), and the association between endoscopic features and histologic findings (as in Study 2).

In Study 1, the most important finding was that there was no significant difference between patients with and without La deposition in the gastric mucosa in terms of blood test findings. In addition, there was no significant difference between the patients taking LaC and those not taking LaC. The reason why the dialysis period is long in the LaC-taking patients was that patients who were on dialysis for longer periods were more likely to develop hyperphosphatemia and be required to take LaC. These results show that gastric La deposition may not cause clinical problems and LaC might be a safe treatment for hyperphosphatasemia in patients with chronic kidney disease on hemodialysis even if La deposits occur in the gastric mucosa. It was revealed that the total dose of LaC was significantly correlated with La deposition in the gastric mucosa. Although our result was similar to our previous report, this study differs in that the daily dose of LaC was not related to La deposition [3]. We had predicted that the daily dose might not significantly affect La deposition when the period of LaC administration was longer. In addition, concomitant drugs (PPIs, H2RAs, NSAIDs, and gastric mucoprotective drugs) did not affect the deposition of La in the gastric mucosa. Although Shitomi et al. reported that the histological finding of *Helicobacter pylori* infection might decrease the deposition of La [5], Ban et al. reported that the lesions associated with *Helicobacter pylori* infection, such as intestinal metaplasia, regenerative changes, and foveolar hyperplasia, were more frequently observed in mucosa with large quantities of La deposits [6, 7]. Given that our present study also showed that the range of atrophic changes in the gastric mucosa was not associated with La deposition, *Helicobacter pylori* infection may not be associated with the deposition of La in the gastric mucosa.

Study 2 revealed that the gastric endoscopic findings of La deposition reflected the infiltrated histiocytes, not the La deposition itself. The appearance of histiocytes must depend on the amount of La deposition, which acts as foreign matter to which histiocytes react. We suggest that the whitish lesions of La deposition reflect the existence of macrophages, similar to gastric xanthoma. La deposition was also detected from non-whitish normal mucosa. In summary, the specific features of La deposition reflect the body’s normal reaction to foreign bodies. However, the reason the histiocytic infiltration was divided clearly into two groups (less than 30% and more than 80%) is uncertain.

Although gastric La deposition may not cause clinical problems and LaC might be safe for hyperphosphatasemia in patients with chronic kidney disease on hemodialysis, it has been reported that several side effects of gastrointestinal symptoms have been associated with LaC, such as nausea, vomiting, and constipation [9]. These symptoms might be caused by gastrointestinal motility dysfunction. As it has been reported that functional dyspepsia is caused, in part, by *Helicobacter pylori* infection [10], the gastrointestinal symptoms in hemodialysis patients taking LaC might be caused by La deposition even if there is no organic lesion. Although it has been reported in a pharmacological safety study of LaC that it has no significant effects on gastric emptying and intestinal transit time at high doses [11, 12], an association between La deposition in the gastric mucosa has been loosely associated with gastric peristalsis. Because the present study did not investigate the relationship between the gastrointestinal symptoms and motility function, we should plan the next study to investigate gastric motility function in hemodialysis patients undergoing LaC treatment.

The present study had some limitations. First, the sample size was small, as this was a single-center retrospective study. In addition, there was some missing blood test data. Second, there may have been patients with gastric La deposition in the La-negative group in Study 1 because Study 2 revealed that La deposition was sometimes detected on biopsy specimens from normal gastric mucosa. Third, although La is often deposited at the duodenal mucosa [8], we did not evaluate duodenal La deposition in the present study because of the low number of subjects or biopsy specimens. There might be a relationship between duodenal La deposition and some background factors. Fourth, in the histological assessment of histiocytic infiltration used in Study 2, it was difficult to remove the subjectivity of the pathologists.

## Conclusion

We demonstrated that La deposition in the gastric mucosa depended on the total dose of LaC and was not affected by any background factors. The specific endoscopic features of La deposition are associated with the infiltration of histiocytes, which represents the body’s normal response to foreign bodies.

## Data Availability

The datasets used and analyzed during the current study will be available from the corresponding author on reasonable request.

## Abbreviations

La: Lanthanum
LaC: Lanthanum carbonate
NSAID: Non-steroidal anti-inflammatory drug
PPI: Proton pump inhibitor
H2RA: Histamine H2 receptor antagonist
CI: Confidence interval
Ca: Calcium
IP: Inorganic phosphorus
TP: Total protein
HDL-cholesterol: High density lipoprotein-cholesterol
WBC: White blood cell
